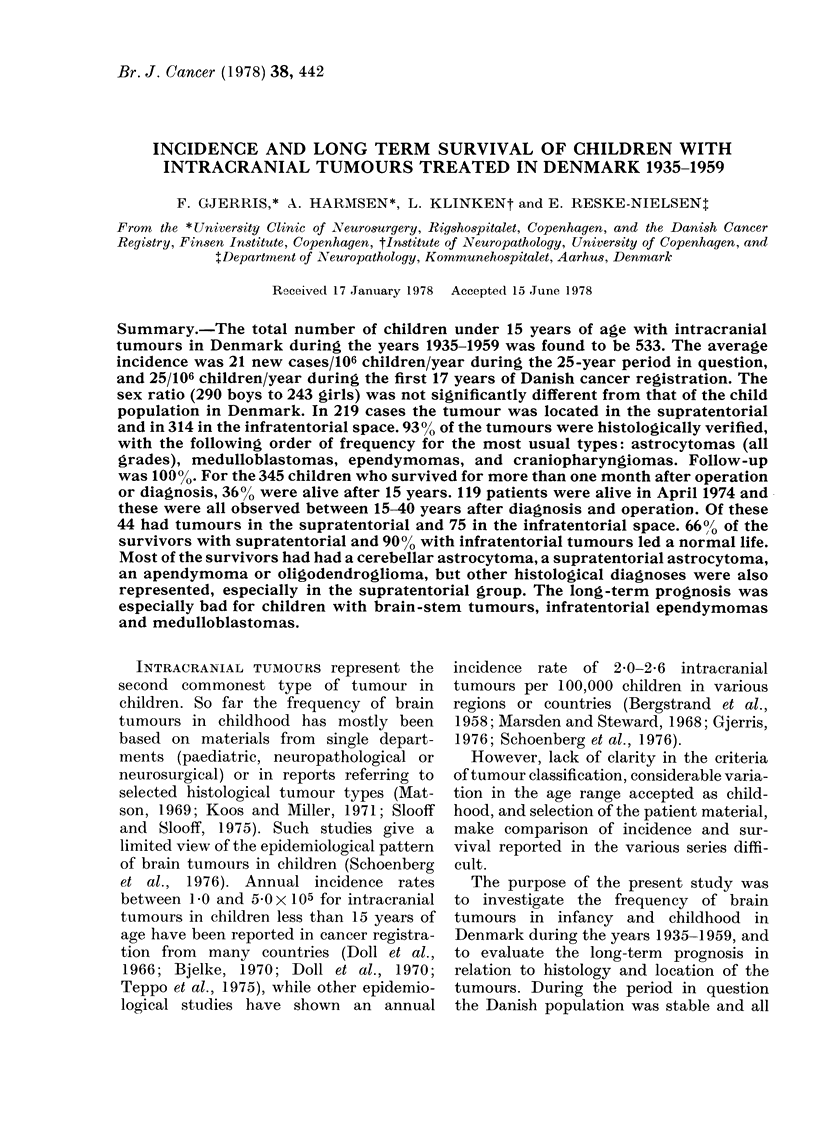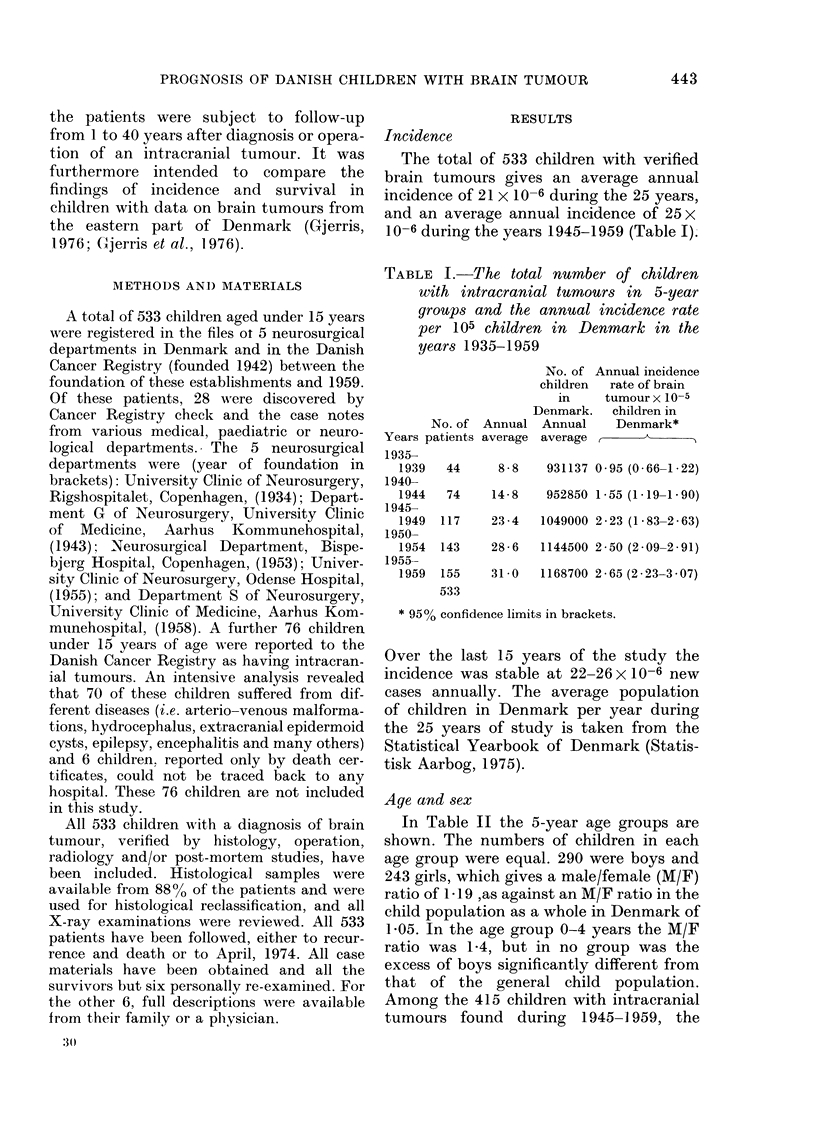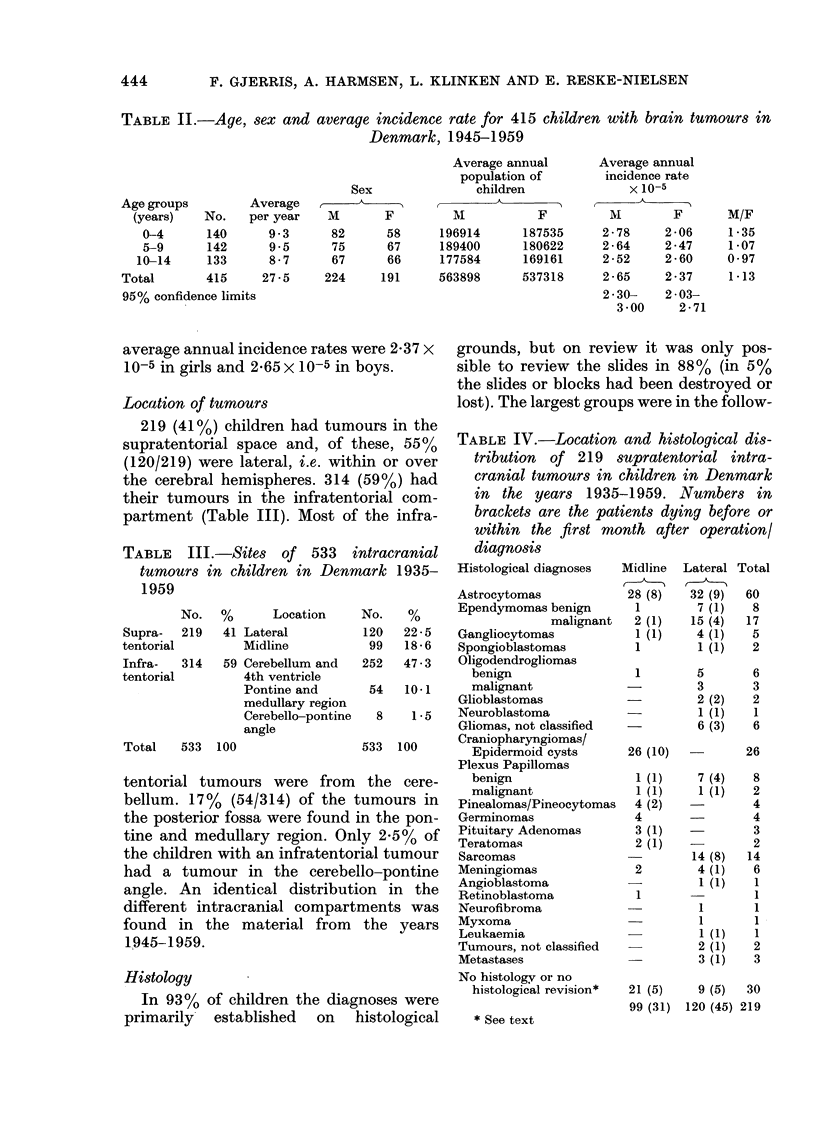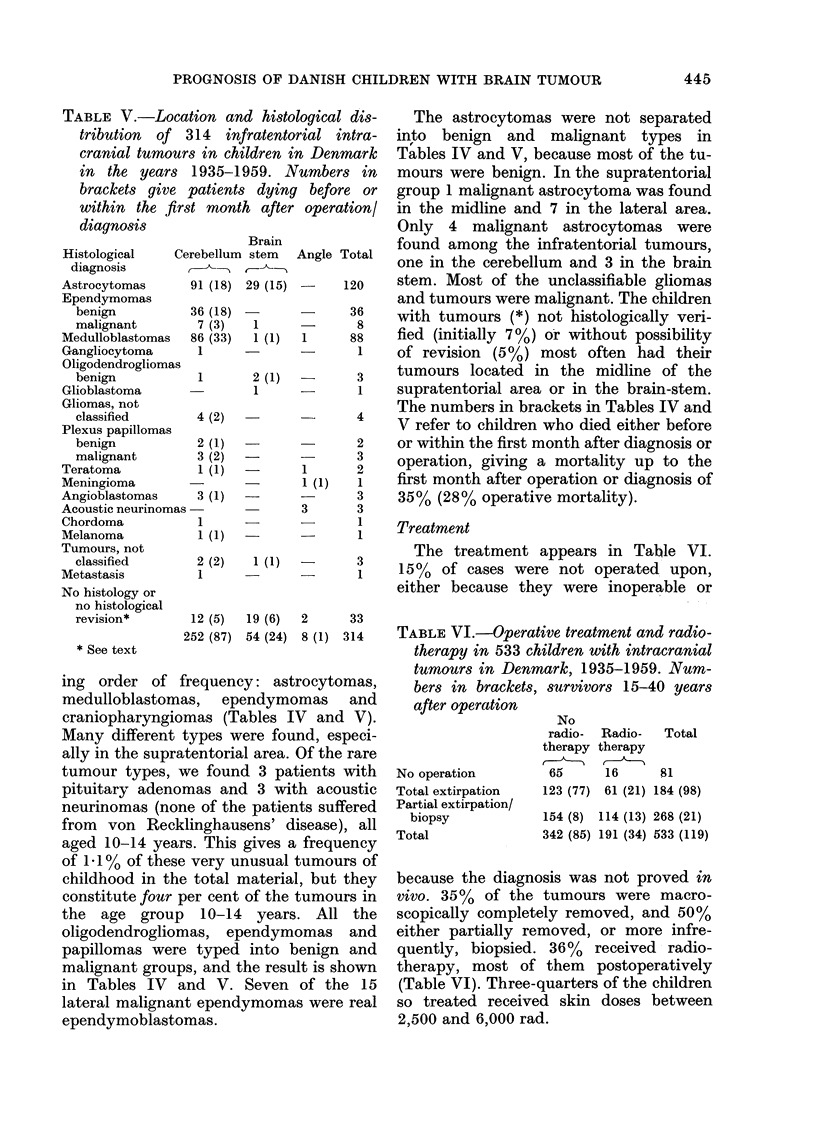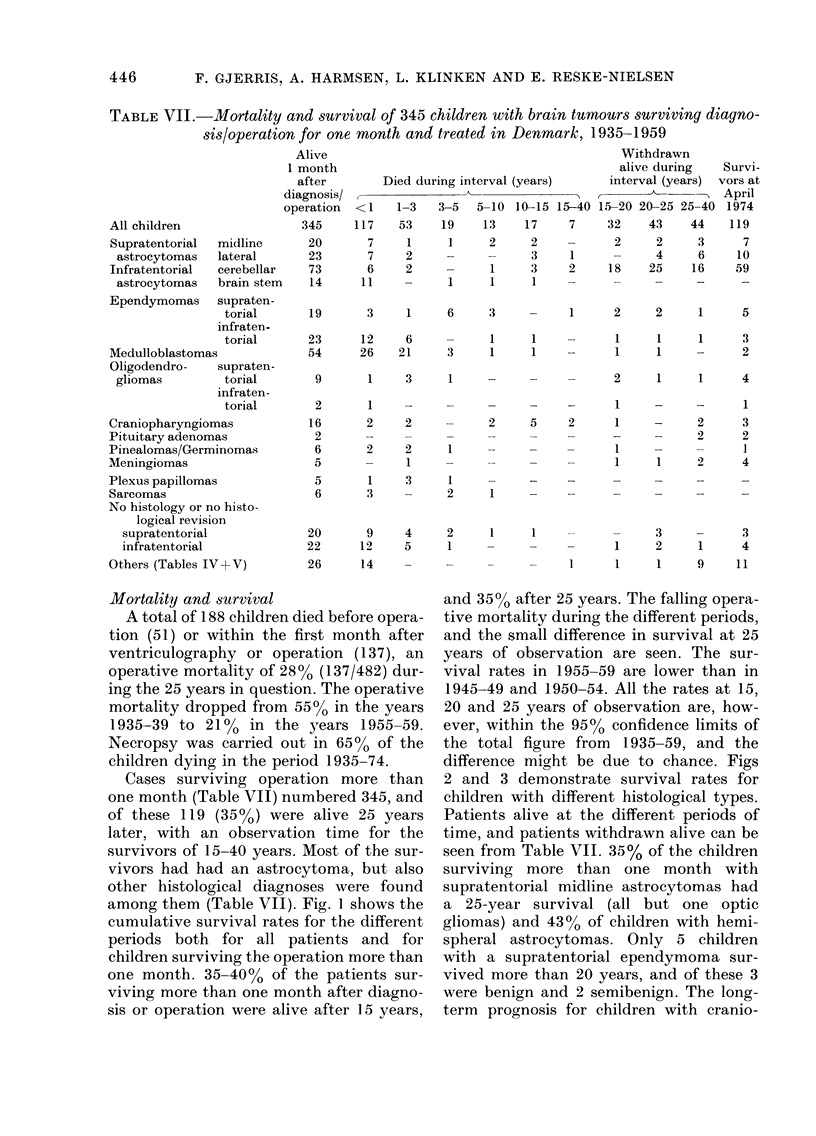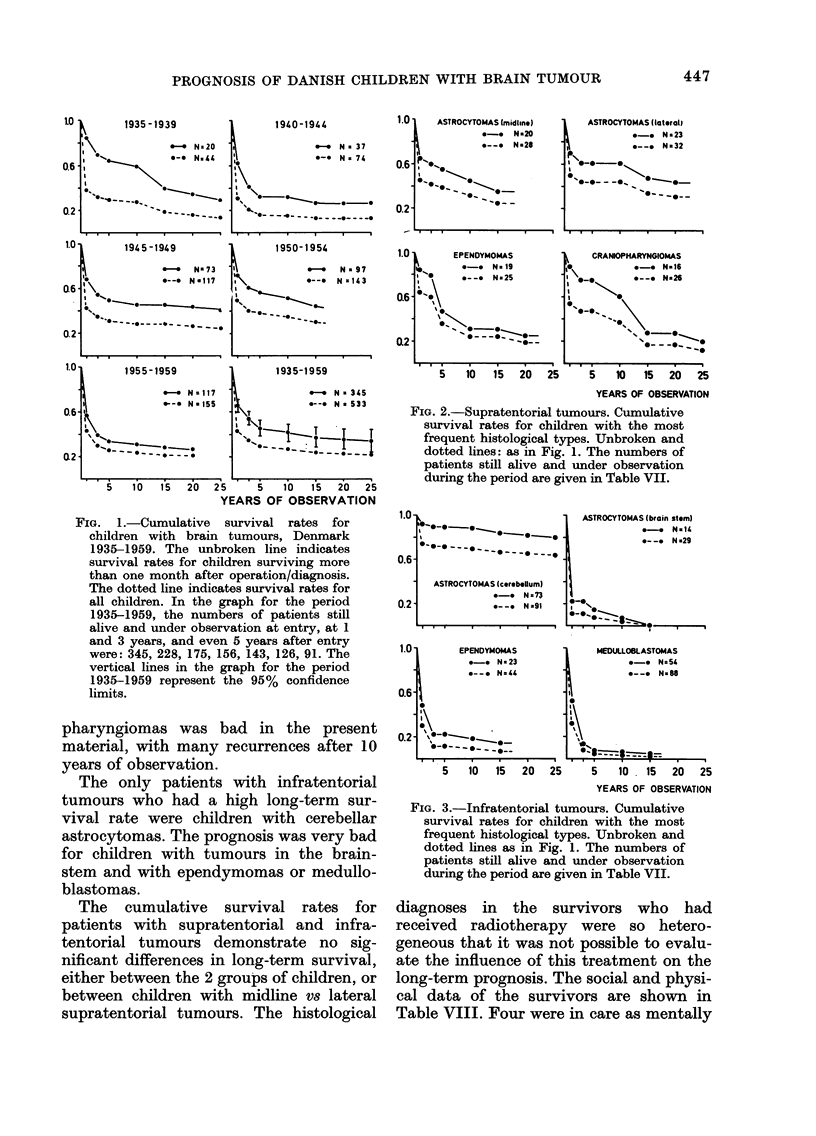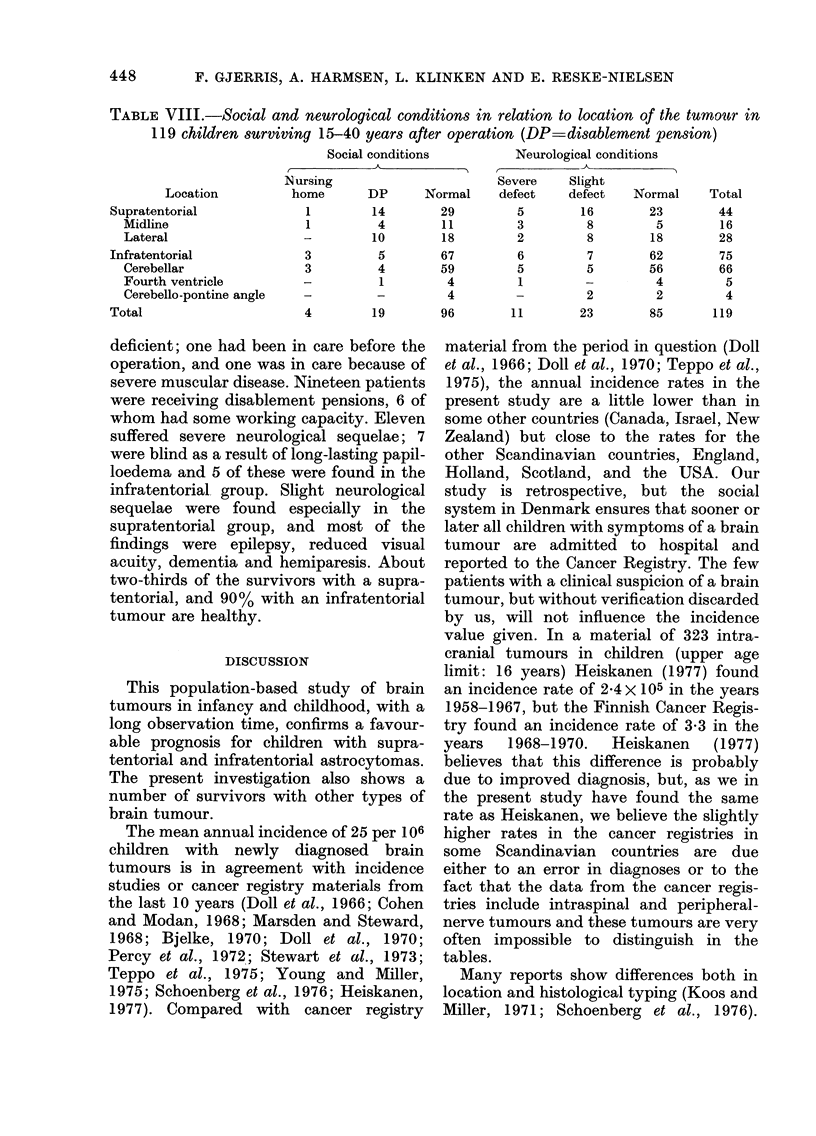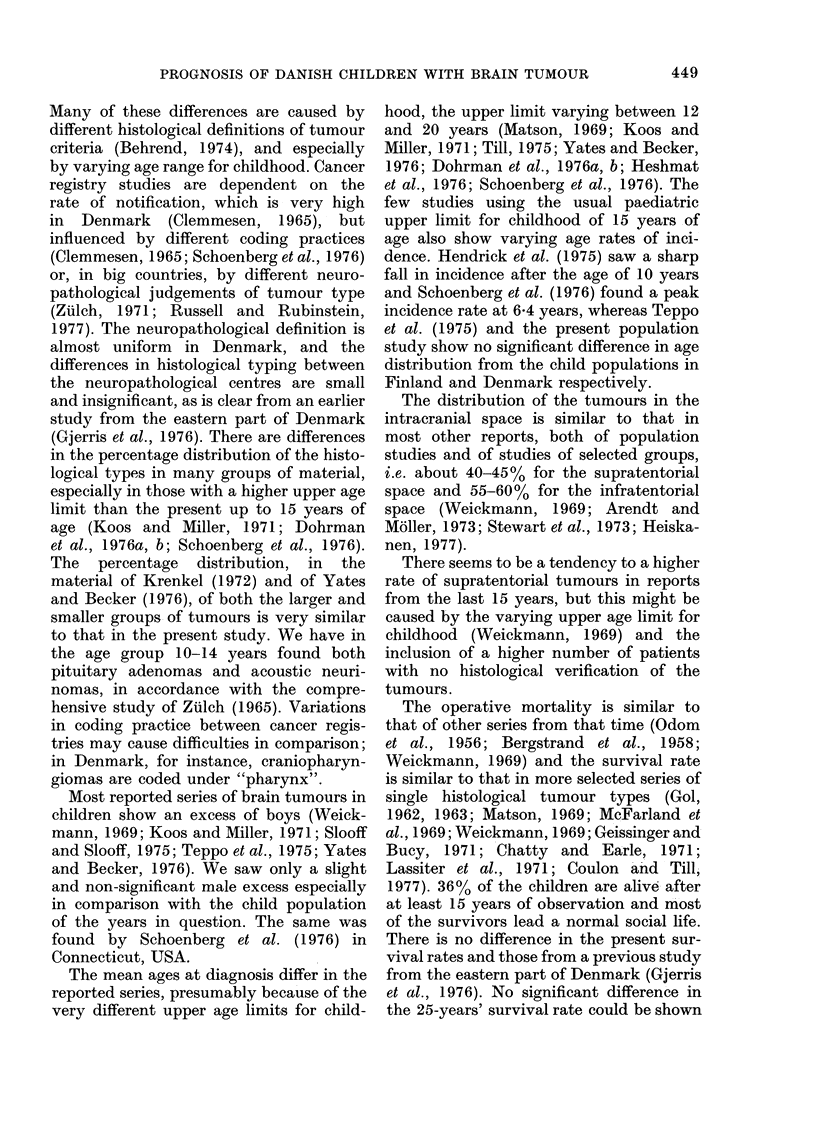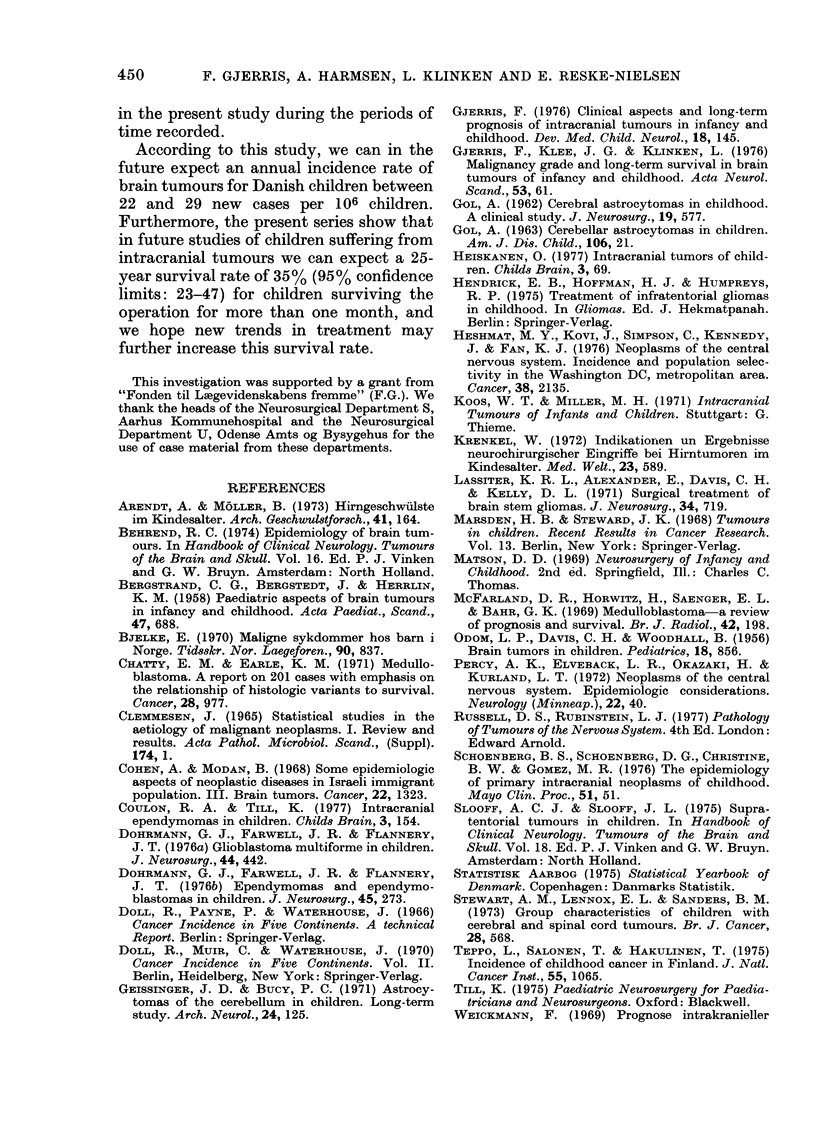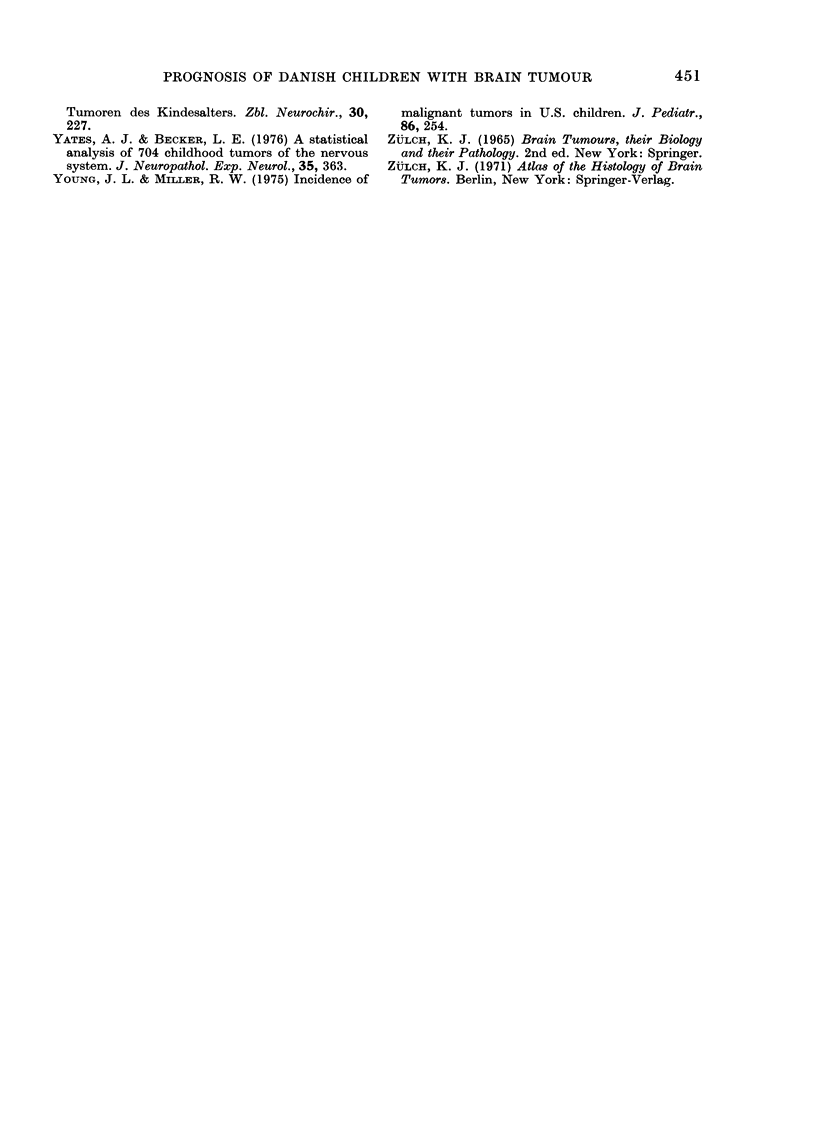# Incidence and long-term survival of children with intracranial tumours treated in Denmark 1935-1959

**DOI:** 10.1038/bjc.1978.227

**Published:** 1978-09

**Authors:** F. Gjerris, A. Harmsen, L. Klinken, E. Reske-Nielsen

## Abstract

The total number of children under 15 years of age with intracranial tumours in Denmark during the years 1935-1959 was found to be 533. The average incidence was 21 new cases/10^6^ children/year during the 25-year period in question, and 25/10^6^ children/year during the first 17 years of Danish cancer registration. The sex ratio (290 boys to 243 girls) was not significantly different from that of the child population in Denmark. In 219 cases the tumour was located in the supratentorial and in 314 in the infratentorial space. 93% of the tumours were histologically verified, with the following order of frequency for the most usual types: astrocytomas (all grades), medulloblastomas, ependymomas, and craniopharyngiomas. Follow-up was 100%. For the 345 children who survived for more than one month after operation or diagnosis, 36% were alive after 15 years. 119 patients were alive in April 1974 and these were all observed between 15-40 years after diagnosis and operation. Of these 44 had tumours in the supratentorial and 75 in the infratentorial space. 66% of the survivors with supratentorial and 90% with infratentorial tumours led a normal life. Most of the survivors had had a cerebellar astrocytoma, a supratentorial astrocytoma, an apendymoma or oligodendroglioma, but other histological diagnoses were also represented, especially in the supratentorial group. The long-term prognosis was especially bad for children with brain-stem tumours, infratentorial ependymomas and medulloblastomas.


*Br. J. Cancer* (1978) **38**, 442

### INCIDENCE AND LONG TERM SURVIVAL OF CHILDREN WITH INTRACRANIAL TUMOURS TREATED IN DENMARK 1935–1959

F. GJERRIS,* A. HARMSEN*, L. KLINKEN† and E. RESKE-NIELSEN‡

*From the *University Clinic of Neurosurgery, Rigshospitalet, Copenhagen, and the Danish Cancer Registry, Finsen Institute, Copenhagen, †Institute of Neuropathology, University of Copenhagen, and ‡Department of Neuropathology, Kommunehospitalet, Aarhus, Denmark*

Received 17 January 1978 Accepted 15 June 1978

**Summary.—The total number of children under 15 years of age with intracranial tumours in Denmark during the years 1935–1959 was found to be 533. The average incidence was 21 new cases/$10^6$ children/year during the 25-year period in question, and 25/$10^6$ children/year during the first 17 years of Danish cancer registration. The sex ratio (290 boys to 243 girls) was not significantly different from that of the child population in Denmark. In 219 cases the tumour was located in the supratentorial and in 314 in the infratentorial space. 93% of the tumours were histologically verified, with the following order of frequency for the most usual types: astrocytomas (all grades), medulloblastomas, ependymomas, and craniopharyngiomas. Follow-up was 100%. For the 345 children who survived for more than one month after operation or diagnosis, 36% were alive after 15 years. 119 patients were alive in April 1974 and these were all observed between 15–40 years after diagnosis and operation. Of these 44 had tumours in the supratentorial and 75 in the infratentorial space. 66% of the survivors with supratentorial and 90% with infratentorial tumours led a normal life. Most of the survivors had had a cerebellar astrocytoma, a supratentorial astrocytoma, an apendymoma or oligodendroglioma, but other histological diagnoses were also represented, especially in the supratentorial group. The long-term prognosis was especially bad for children with brain-stem tumours, infratentorial ependymomas and medulloblastomas.**

INTRACRANIAL TUMOURS represent the second commonest type of tumour in children. So far the frequency of brain tumours in childhood has mostly been based on materials from single departments (paediatric, neuropathological or neurosurgical) or in reports referring to selected histological tumour types (Matson, 1969; Koos and Miller, 1971; Slooff and Slooff, 1975). Such studies give a limited view of the epidemiological pattern of brain tumours in children (Schoenberg *et al.*, 1976). Annual incidence rates between 1·0 and $5{\cdot}0 \times 10^5$ for intracranial tumours in children less than 15 years of age have been reported in cancer registration from many countries (Doll *et al.*, 1966; Bjelke, 1970; Doll *et al.*, 1970; Teppo *et al.*, 1975), while other epidemiological studies have shown an annual incidence rate of 2·0–2·6 intracranial tumours per 100,000 children in various regions or countries (Bergstrand *et al.*, 1958; Marsden and Steward, 1968; Gjerris, 1976; Schoenberg *et al.*, 1976).

However, lack of clarity in the criteria of tumour classification, considerable variation in the age range accepted as childhood, and selection of the patient material, make comparison of incidence and survival reported in the various series difficult.

The purpose of the present study was to investigate the frequency of brain tumours in infancy and childhood in Denmark during the years 1935–1959, and to evaluate the long-term prognosis in relation to histology and location of the tumours. During the period in question the Danish population was stable and all the patients were subject to follow-up from 1 to 40 years after diagnosis or operation of an intracranial tumour. It was furthermore intended to compare the findings of incidence and survival in children with data on brain tumours from the eastern part of Denmark (Gjerris, 1976; Gjerris *et al.*, 1976).

### METHODS AND MATERIALS

A total of 533 children aged under 15 years were registered in the files of 5 neurosurgical departments in Denmark and in the Danish Cancer Registry (founded 1942) between the foundation of these establishments and 1959. Of these patients, 28 were discovered by Cancer Registry check and the case notes from various medical, paediatric or neurological departments. The 5 neurosurgical departments were (year of foundation in brackets): University Clinic of Neurosurgery, Rigshospitalet, Copenhagen, (1934); Department G of Neurosurgery, University Clinic of Medicine, Aarhus Kommunehospital, (1943); Neurosurgical Department, Bispebjerg Hospital, Copenhagen, (1953); University Clinic of Neurosurgery, Odense Hospital, (1955); and Department S of Neurosurgery, University Clinic of Medicine, Aarhus Kommunehospital, (1958). A further 76 children under 15 years of age were reported to the Danish Cancer Registry as having intracranial tumours. An intensive analysis revealed that 70 of these children suffered from different diseases (*i.e.* arterio–venous malformations, hydrocephalus, extracranial epidermoid cysts, epilepsy, encephalitis and many others) and 6 children, reported only by death certificates, could not be traced back to any hospital. These 76 children are not included in this study.

All 533 children with a diagnosis of brain tumour, verified by histology, operation, radiology and/or post-mortem studies, have been included. Histological samples were available from 88% of the patients and were used for histological reclassification, and all X-ray examinations were reviewed. All 533 patients have been followed, either to recurrence and death or to April, 1974. All case materials have been obtained and all the survivors but six personally re-examined. For the other 6, full descriptions were available from their family or a physician.

### RESULTS

### *Incidence*

The total of 533 children with verified brain tumours gives an average annual incidence of $21 \times 10^{-6}$ during the 25 years, and an average annual incidence of $25 \times 10^{-6}$ during the years 1945–1959 (Table I).

TABLE I.—*The total number of children with intracranial tumours in 5-year groups and the annual incidence rate per* $10^5$ *children in Denmark in the years* 1935–1959

| Years | No. of patients | Annual average | No. of children in Denmark. Annual average | Annual incidence rate of brain tumour $\times 10^{-5}$ children in Denmark* |
|---|---|---|---|---|
| 1935–1939 | 44 | 8·8 | 931137 | 0·95 (0·66–1·22) |
| 1940–1944 | 74 | 14·8 | 952850 | 1·55 (1·19–1·90) |
| 1945–1949 | 117 | 23·4 | 1049000 | 2·23 (1·83–2·63) |
| 1950–1954 | 143 | 28·6 | 1144500 | 2·50 (2·09–2·91) |
| 1955–1959 | 155 | 31·0 | 1168700 | 2·65 (2·23–3·07) |
| | 533 | | | |

* 95% confidence limits in brackets.

Over the last 15 years of the study the incidence was stable at $22–26 \times 10^{-6}$ new cases annually. The average population of children in Denmark per year during the 25 years of study is taken from the Statistical Yearbook of Denmark (Statistisk Aarbog, 1975).

### *Age and sex*

In Table II the 5-year age groups are shown. The numbers of children in each age group were equal. 290 were boys and 243 girls, which gives a male/female (M/F) ratio of 1·19 ,as against an M/F ratio in the child population as a whole in Denmark of 1·05. In the age group 0–4 years the M/F ratio was 1·4, but in no group was the excess of boys significantly different from that of the general child population. Among the 415 children with intracranial tumours found during 1945–1959, the

TABLE II.—*Age, sex and average incidence rate for 415 children with brain tumours in Denmark, 1945–1959*

| Age groups (years) | No. | Average per year | Sex | | Average annual population of children | | Average annual incidence rate $\times 10^{-5}$ | | |
|---|---|---|---|---|---|---|---|---|---|
| | | | M | F | M | F | M | F | M/F |
| 0–4 | 140 | 9·3 | 82 | 58 | 196914 | 187535 | 2·78 | 2·06 | 1·35 |
| 5–9 | 142 | 9·5 | 75 | 67 | 189400 | 180622 | 2·64 | 2·47 | 1·07 |
| 10–14 | 133 | 8·7 | 67 | 66 | 177584 | 169161 | 2·52 | 2·60 | 0·97 |
| Total | 415 | 27·5 | 224 | 191 | 563898 | 537318 | 2·65 | 2·37 | 1·13 |
| 95% confidence limits | | | | | | | 2·30–3·00 | 2·03–2·71 | |

average annual incidence rates were $2{\cdot}37 \times 10^{-5}$ in girls and $2{\cdot}65 \times 10^{-5}$ in boys.

*Location of tumours*

219 (41%) children had tumours in the supratentorial space and, of these, 55% (120/219) were lateral, *i.e.* within or over the cerebral hemispheres. 314 (59%) had their tumours in the infratentorial compartment (Table III). Most of the infratentorial tumours were from the cerebellum. 17% (54/314) of the tumours in the posterior fossa were found in the pontine and medullary region. Only 2·5% of the children with an infratentorial tumour had a tumour in the cerebello–pontine angle. An identical distribution in the different intracranial compartments was found in the material from the years 1945–1959.

TABLE III.—*Sites of 533 intracranial tumours in children in Denmark* 1935–1959

| | No. | % | Location | No. | % |
|---|---|---|---|---|---|
| Supra-tentorial | 219 | 41 | Lateral | 120 | 22·5 |
| | | | Midline | 99 | 18·6 |
| Infra-tentorial | 314 | 59 | Cerebellum and 4th ventricle | 252 | 47·3 |
| | | | Pontine and medullary region | 54 | 10·1 |
| | | | Cerebello–pontine angle | 8 | 1·5 |
| Total | 533 | 100 | | 533 | 100 |

*Histology*

In 93% of children the diagnoses were primarily established on histological grounds, but on review it was only possible to review the slides in 88% (in 5% the slides or blocks had been destroyed or lost). The largest groups were in the follow-

TABLE IV.—*Location and histological distribution of 219 supratentorial intracranial tumours in children in Denmark in the years 1935–1959. Numbers in brackets are the patients dying before or within the first month after operation/diagnosis*

| Histological diagnoses | Midline | Lateral | Total |
|---|---|---|---|
| Astrocytomas | 28 (8) | 32 (9) | 60 |
| Ependymomas benign | 1 | 7 (1) | 8 |
| malignant | 2 (1) | 15 (4) | 17 |
| Gangliocytomas | 1 (1) | 4 (1) | 5 |
| Spongioblastomas | 1 | 1 (1) | 2 |
| Oligodendrogliomas | | | |
| benign | 1 | 5 | 6 |
| malignant | — | 3 | 3 |
| Glioblastomas | — | 2 (2) | 2 |
| Neuroblastoma | — | 1 (1) | 1 |
| Gliomas, not classified | — | 6 (3) | 6 |
| Craniopharyngiomas/ Epidermoid cysts | 26 (10) | — | 26 |
| Plexus Papillomas | | | |
| benign | 1 (1) | 7 (4) | 8 |
| malignant | 1 (1) | 1 (1) | 2 |
| Pinealomas/Pineocytomas | 4 (2) | — | 4 |
| Germinomas | 4 | — | 4 |
| Pituitary Adenomas | 3 (1) | — | 3 |
| Teratomas | 2 (1) | — | 2 |
| Sarcomas | — | 14 (8) | 14 |
| Meningiomas | 2 | 4 (1) | 6 |
| Angioblastoma | — | 1 (1) | 1 |
| Retinoblastoma | 1 | — | 1 |
| Neurofibroma | — | 1 | 1 |
| Myxoma | — | 1 | 1 |
| Leukaemia | — | 1 (1) | 1 |
| Tumours, not classified | — | 2 (1) | 2 |
| Metastases | — | 3 (1) | 3 |
| No histology or no histological revision* | 21 (5) | 9 (5) | 30 |
| | 99 (31) | 120 (45) | 219 |

* See text

TABLE V.—*Location and histological distribution of* 314 *infratentorial intracranial tumours in children in Denmark in the years* 1935–1959. *Numbers in brackets give patients dying before or within the first month after operation/diagnosis*

| Histological diagnosis | Cerebellum | Brain stem | Angle | Total |
|---|---|---|---|---|
| Astrocytomas | 91 (18) | 29 (15) | — | 120 |
| Ependymomas | | | | |
| benign | 36 (18) | — | — | 36 |
| malignant | 7 (3) | 1 | — | 8 |
| Medulloblastomas | 86 (33) | 1 (1) | 1 | 88 |
| Gangliocytoma | 1 | — | — | 1 |
| Oligodendrogliomas | | | | |
| benign | 1 | 2 (1) | — | 3 |
| Glioblastoma | — | 1 | — | 1 |
| Gliomas, not classified | 4 (2) | — | — | 4 |
| Plexus papillomas | | | | |
| benign | 2 (1) | — | — | 2 |
| malignant | 3 (2) | — | — | 3 |
| Teratoma | 1 (1) | — | 1 | 2 |
| Meningioma | — | — | 1 (1) | 1 |
| Angioblastomas | 3 (1) | — | — | 3 |
| Acoustic neurinomas | — | — | 3 | 3 |
| Chordoma | 1 | — | — | 1 |
| Melanoma | 1 (1) | — | — | 1 |
| Tumours, not classified | 2 (2) | 1 (1) | — | 3 |
| Metastasis | 1 | — | — | 1 |
| No histology or no histological revision* | 12 (5) | 19 (6) | 2 | 33 |
| | 252 (87) | 54 (24) | 8 (1) | 314 |

* See text ing order of frequency: astrocytomas, medulloblastomas, ependymomas and craniopharyngiomas (Tables IV and V). Many different types were found, especially in the supratentorial area. Of the rare tumour types, we found 3 patients with pituitary adenomas and 3 with acoustic neurinomas (none of the patients suffered from von Recklinghausens' disease), all aged 10–14 years. This gives a frequency of 1·1% of these very unusual tumours of childhood in the total material, but they constitute *four* per cent of the tumours in the age group 10–14 years. All the oligodendrogliomas, ependymomas and papillomas were typed into benign and malignant groups, and the result is shown in Tables IV and V. Seven of the 15 lateral malignant ependymomas were real ependymoblastomas.

The astrocytomas were not separated into benign and malignant types in Tables IV and V, because most of the tumours were benign. In the supratentorial group 1 malignant astrocytoma was found in the midline and 7 in the lateral area. Only 4 malignant astrocytomas were found among the infratentorial tumours, one in the cerebellum and 3 in the brain stem. Most of the unclassifiable gliomas and tumours were malignant. The children with tumours (*) not histologically verified (initially 7%) or without possibility of revision (5%) most often had their tumours located in the midline of the supratentorial area or in the brain-stem. The numbers in brackets in Tables IV and V refer to children who died either before or within the first month after diagnosis or operation, giving a mortality up to the first month after operation or diagnosis of 35% (28% operative mortality).

### *Treatment*

The treatment appears in Table VI. 15% of cases were not operated upon, either because they were inoperable or because the diagnosis was not proved *in vivo.* 35% of the tumours were macroscopically completely removed, and 50% either partially removed, or more infrequently, biopsied. 36% received radiotherapy, most of them postoperatively (Table VI). Three-quarters of the children so treated received skin doses between 2,500 and 6,000 rad.

TABLE VI.—*Operative treatment and radiotherapy in* 533 *children with intracranial tumours in Denmark,* 1935–1959. *Numbers in brackets, survivors* 15–40 *years after operation*

| | No radiotherapy | Radiotherapy | Total |
|---|---|---|---|
| No operation | 65 | 16 | 81 |
| Total extirpation | 123 (77) | 61 (21) | 184 (98) |
| Partial extirpation/biopsy | 154 (8) | 114 (13) | 268 (21) |
| Total | 342 (85) | 191 (34) | 533 (119) |

TABLE VII.—*Mortality and survival of* 345 *children with brain tumours surviving diagnosis/operation for one month and treated in Denmark,* 1935–1959

| | | Alive 1 month after diagnosis/ operation | Died during interval (years) | | | | | | Withdrawn alive during interval (years) | | | Survivors at April 1974 |
|---|---|---|---|---|---|---|---|---|---|---|---|---|
| | | | <1 | 1–3 | 3–5 | 5–10 | 10–15 | 15–40 | 15–20 | 20–25 | 25–40 | |
| All children | | 345 | 117 | 53 | 19 | 13 | 17 | 7 | 32 | 43 | 44 | 119 |
| Supratentorial | midline | 20 | 7 | 1 | 1 | 2 | 2 | – | 2 | 2 | 3 | 7 |
| astrocytomas | lateral | 23 | 7 | 2 | – | – | 3 | 1 | – | 4 | 6 | 10 |
| Infratentorial | cerebellar | 73 | 6 | 2 | – | 1 | 3 | 2 | 18 | 25 | 16 | 59 |
| astrocytomas | brain stem | 14 | 11 | – | 1 | 1 | 1 | – | – | – | – | – |
| Ependymomas | supratentorial | 19 | 3 | 1 | 6 | 3 | – | 1 | 2 | 2 | 1 | 5 |
| | infratentorial | 23 | 12 | 6 | – | 1 | 1 | – | 1 | 1 | 1 | 3 |
| Medulloblastomas | | 54 | 26 | 21 | 3 | 1 | 1 | – | 1 | 1 | – | 2 |
| Oligodendrogliomas | supratentorial | 9 | 1 | 3 | 1 | – | – | – | 2 | 1 | 1 | 4 |
| | infratentorial | 2 | 1 | – | – | – | – | – | 1 | – | – | 1 |
| Craniopharyngiomas | | 16 | 2 | 2 | – | 2 | 5 | 2 | 1 | – | 2 | 3 |
| Pituitary adenomas | | 2 | – | – | – | – | – | – | – | – | 2 | 2 |
| Pinealomas/Germinomas | | 6 | 2 | 2 | 1 | – | – | – | 1 | – | – | 1 |
| Meningiomas | | 5 | – | 1 | – | – | – | – | 1 | 1 | 2 | 4 |
| Plexus papillomas | | 5 | 1 | 3 | 1 | – | – | – | – | – | – | – |
| Sarcomas | | 6 | 3 | – | 2 | 1 | – | – | – | – | – | – |
| No histology or no histological revision | | | | | | | | | | | | |
| supratentorial | | 20 | 9 | 4 | 2 | 1 | 1 | – | – | 3 | – | 3 |
| infratentorial | | 22 | 12 | 5 | 1 | – | – | – | 1 | 2 | 1 | 4 |
| Others (Tables IV+V) | | 26 | 14 | – | – | – | – | 1 | 1 | 1 | 9 | 11 |

*Mortality and survival*

A total of 188 children died before operation (51) or within the first month after ventriculography or operation (137), an operative mortality of 28% (137/482) during the 25 years in question. The operative mortality dropped from 55% in the years 1935–39 to 21% in the years 1955–59. Necropsy was carried out in 65% of the children dying in the period 1935–74.

Cases surviving operation more than one month (Table VII) numbered 345, and of these 119 (35%) were alive 25 years later, with an observation time for the survivors of 15–40 years. Most of the survivors had had an astrocytoma, but also other histological diagnoses were found among them (Table VII). Fig. 1 shows the cumulative survival rates for the different periods both for all patients and for children surviving the operation more than one month. 35–40% of the patients surviving more than one month after diagnosis or operation were alive after 15 years, and 35% after 25 years. The falling operative mortality during the different periods, and the small difference in survival at 25 years of observation are seen. The survival rates in 1955–59 are lower than in 1945–49 and 1950–54. All the rates at 15, 20 and 25 years of observation are, however, within the 95% confidence limits of the total figure from 1935–59, and the difference might be due to chance. Figs 2 and 3 demonstrate survival rates for children with different histological types. Patients alive at the different periods of time, and patients withdrawn alive can be seen from Table VII. 35% of the children surviving more than one month with supratentorial midline astrocytomas had a 25-year survival (all but one optic gliomas) and 43% of children with hemispheral astrocytomas. Only 5 children with a supratentorial ependymoma survived more than 20 years, and of these 3 were benign and 2 semibenign. The long-term prognosis for children with cranio-

FIG. 1.—Cumulative survival rates for children with brain tumours, Denmark 1935–1959. The unbroken line indicates survival rates for children surviving more than one month after operation/diagnosis. The dotted line indicates survival rates for all children. In the graph for the period 1935–1959, the numbers of patients still alive and under observation at entry, at 1 and 3 years, and even 5 years after entry were: 345, 228, 175, 156, 143, 126, 91. The vertical lines in the graph for the period 1935–1959 represent the 95% confidence limits.

FIG. 2.—Supratentorial tumours. Cumulative survival rates for children with the most frequent histological types. Unbroken and dotted lines: as in Fig. 1. The numbers of patients still alive and under observation during the period are given in Table VII.

FIG. 3.—Infratentorial tumours. Cumulative survival rates for children with the most frequent histological types. Unbroken and dotted lines as in Fig. 1. The numbers of patients still alive and under observation during the period are given in Table VII.

pharyngiomas was bad in the present material, with many recurrences after 10 years of observation.

The only patients with infratentorial tumours who had a high long-term survival rate were children with cerebellar astrocytomas. The prognosis was very bad for children with tumours in the brainstem and with ependymomas or medulloblastomas.

The cumulative survival rates for patients with supratentorial and infratentorial tumours demonstrate no significant differences in long-term survival, either between the 2 groups of children, or between children with midline *vs* lateral supratentorial tumours. The histological diagnoses in the survivors who had received radiotherapy were so heterogeneous that it was not possible to evaluate the influence of this treatment on the long-term prognosis. The social and physical data of the survivors are shown in Table VIII. Four were in care as mentally

TABLE VIII.—*Social and neurological conditions in relation to location of the tumour in 119 children surviving 15–40 years after operation (DP=disablement pension)*

| Location | Social conditions: Nursing home | DP | Normal | Neurological conditions: Severe defect | Slight defect | Normal | Total |
|---|---|---|---|---|---|---|---|
| Supratentorial | 1 | 14 | 29 | 5 | 16 | 23 | 44 |
| Midline | 1 | 4 | 11 | 3 | 8 | 5 | 16 |
| Lateral | – | 10 | 18 | 2 | 8 | 18 | 28 |
| Infratentorial | 3 | 5 | 67 | 6 | 7 | 62 | 75 |
| Cerebellar | 3 | 4 | 59 | 5 | 5 | 56 | 66 |
| Fourth ventricle | – | 1 | 4 | 1 | – | 4 | 5 |
| Cerebello-pontine angle | – | – | 4 | – | 2 | 2 | 4 |
| Total | 4 | 19 | 96 | 11 | 23 | 85 | 119 |

deficient; one had been in care before the operation, and one was in care because of severe muscular disease. Nineteen patients were receiving disablement pensions, 6 of whom had some working capacity. Eleven suffered severe neurological sequelae; 7 were blind as a result of long-lasting papilloedema and 5 of these were found in the infratentorial group. Slight neurological sequelae were found especially in the supratentorial group, and most of the findings were epilepsy, reduced visual acuity, dementia and hemiparesis. About two-thirds of the survivors with a supratentorial, and 90% with an infratentorial tumour are healthy.

### DISCUSSION

This population-based study of brain tumours in infancy and childhood, with a long observation time, confirms a favourable prognosis for children with supratentorial and infratentorial astrocytomas. The present investigation also shows a number of survivors with other types of brain tumour.

The mean annual incidence of 25 per $10^6$ children with newly diagnosed brain tumours is in agreement with incidence studies or cancer registry materials from the last 10 years (Doll *et al.*, 1966; Cohen and Modan, 1968; Marsden and Steward, 1968; Bjelke, 1970; Doll *et al.*, 1970; Percy *et al.*, 1972; Stewart *et al.*, 1973; Teppo *et al.*, 1975; Young and Miller, 1975; Schoenberg *et al.*, 1976; Heiskanen, 1977). Compared with cancer registry material from the period in question (Doll *et al.*, 1966; Doll *et al.*, 1970; Teppo *et al.*, 1975), the annual incidence rates in the present study are a little lower than in some other countries (Canada, Israel, New Zealand) but close to the rates for the other Scandinavian countries, England, Holland, Scotland, and the USA. Our study is retrospective, but the social system in Denmark ensures that sooner or later all children with symptoms of a brain tumour are admitted to hospital and reported to the Cancer Registry. The few patients with a clinical suspicion of a brain tumour, but without verification discarded by us, will not influence the incidence value given. In a material of 323 intracranial tumours in children (upper age limit: 16 years) Heiskanen (1977) found an incidence rate of $2{\cdot}4 \times 10^5$ in the years 1958–1967, but the Finnish Cancer Registry found an incidence rate of 3·3 in the years 1968–1970. Heiskanen (1977) believes that this difference is probably due to improved diagnosis, but, as we in the present study have found the same rate as Heiskanen, we believe the slightly higher rates in the cancer registries in some Scandinavian countries are due either to an error in diagnoses or to the fact that the data from the cancer registries include intraspinal and peripheral-nerve tumours and these tumours are very often impossible to distinguish in the tables.

Many reports show differences both in location and histological typing (Koos and Miller, 1971; Schoenberg *et al.*, 1976).

Many of these differences are caused by different histological definitions of tumour criteria (Behrend, 1974), and especially by varying age range for childhood. Cancer registry studies are dependent on the rate of notification, which is very high in Denmark (Clemmesen, 1965), but influenced by different coding practices (Clemmesen, 1965; Schoenberg *et al.*, 1976) or, in big countries, by different neuropathological judgements of tumour type (Zülch, 1971; Russell and Rubinstein, 1977). The neuropathological definition is almost uniform in Denmark, and the differences in histological typing between the neuropathological centres are small and insignificant, as is clear from an earlier study from the eastern part of Denmark (Gjerris *et al.*, 1976). There are differences in the percentage distribution of the histological types in many groups of material, especially in those with a higher upper age limit than the present up to 15 years of age (Koos and Miller, 1971; Dohrman *et al.*, 1976*a*, *b*; Schoenberg *et al.*, 1976). The percentage distribution, in the material of Krenkel (1972) and of Yates and Becker (1976), of both the larger and smaller groups of tumours is very similar to that in the present study. We have in the age group 10–14 years found both pituitary adenomas and acoustic neurinomas, in accordance with the comprehensive study of Zülch (1965). Variations in coding practice between cancer registries may cause difficulties in comparison; in Denmark, for instance, craniopharyngiomas are coded under "pharynx".

Most reported series of brain tumours in children show an excess of boys (Weickmann, 1969; Koos and Miller, 1971; Slooff and Slooff, 1975; Teppo *et al.*, 1975; Yates and Becker, 1976). We saw only a slight and non-significant male excess especially in comparison with the child population of the years in question. The same was found by Schoenberg *et al.* (1976) in Connecticut, USA.

The mean ages at diagnosis differ in the reported series, presumably because of the very different upper age limits for childhood, the upper limit varying between 12 and 20 years (Matson, 1969; Koos and Miller, 1971; Till, 1975; Yates and Becker, 1976; Dohrman *et al.*, 1976*a*, *b*; Heshmat *et al.*, 1976; Schoenberg *et al.*, 1976). The few studies using the usual paediatric upper limit for childhood of 15 years of age also show varying age rates of incidence. Hendrick *et al.* (1975) saw a sharp fall in incidence after the age of 10 years and Schoenberg *et al.* (1976) found a peak incidence rate at 6·4 years, whereas Teppo *et al.* (1975) and the present population study show no significant difference in age distribution from the child populations in Finland and Denmark respectively.

The distribution of the tumours in the intracranial space is similar to that in most other reports, both of population studies and of studies of selected groups, *i.e.* about 40–45% for the supratentorial space and 55–60% for the infratentorial space (Weickmann, 1969; Arendt and Möller, 1973; Stewart *et al.*, 1973; Heiskanen, 1977).

There seems to be a tendency to a higher rate of supratentorial tumours in reports from the last 15 years, but this might be caused by the varying upper age limit for childhood (Weickmann, 1969) and the inclusion of a higher number of patients with no histological verification of the tumours.

The operative mortality is similar to that of other series from that time (Odom *et al.*, 1956; Bergstrand *et al.*, 1958; Weickmann, 1969) and the survival rate is similar to that in more selected series of single histological tumour types (Gol, 1962, 1963; Matson, 1969; McFarland *et al.*, 1969; Weickmann, 1969; Geissinger and Bucy, 1971; Chatty and Earle, 1971; Lassiter *et al.*, 1971; Coulon and Till, 1977). 36% of the children are alive after at least 15 years of observation and most of the survivors lead a normal social life. There is no difference in the present survival rates and those from a previous study from the eastern part of Denmark (Gjerris *et al.*, 1976). No significant difference in the 25-years' survival rate could be shown in the present study during the periods of time recorded.

According to this study, we can in the future expect an annual incidence rate of brain tumours for Danish children between 22 and 29 new cases per $10^6$ children. Furthermore, the present series show that in future studies of children suffering from intracranial tumours we can expect a 25-year survival rate of 35% (95% confidence limits: 23–47) for children surviving the operation for more than one month, and we hope new trends in treatment may further increase this survival rate.

This investigation was supported by a grant from "Fonden til Lægevidenskabens fremme" (F.G.). We thank the heads of the Neurosurgical Department S, Aarhus Kommunehospital and the Neurosurgical Department U, Odense Amts og Bysygehus for the use of case material from these departments.

### REFERENCES

ARENDT, A. & MÖLLER, B. (1973) Hirngeschwülste im Kindesalter. *Arch. Geschwulstforsch.*, **41**, 164.

BEHREND, R. C. (1974) Epidemiology of brain tumours. In *Handbook of Clinical Neurology. Tumours of the Brain and Skull.* Vol. 16. Ed. P. J. Vinken and G. W. Bruyn. Amsterdam: North Holland.

BERGSTRAND, C. G., BERGSTEDT, J. & HERRLIN, K. M. (1958) Paediatric aspects of brain tumours in infancy and childhood. *Acta Paediat., Scand.*, **47**, 688.

BJELKE, E. (1970) Maligne sykdommer hos barn i Norge. *Tidsskr. Nor. Laegeforen.*, **90**, 837.

CHATTY, E. M. & EARLE, K. M. (1971) Medulloblastoma. A report on 201 cases with emphasis on the relationship of histologic variants to survival. *Cancer*, **28**, 977.

CLEMMESEN, J. (1965) Statistical studies in the aetiology of malignant neoplasms. I. Review and results. *Acta Pathol. Microbiol. Scand.*, (Suppl). **174**, 1.

COHEN, A. & MODAN, B. (1968) Some epidemiologic aspects of neoplastic diseases in Israeli immigrant population. III. Brain tumors. *Cancer*, **22**, 1323.

COULON, R. A. & TILL, K. (1977) Intracranial ependymomas in children. *Childs Brain*, **3**, 154.

DOHRMANN, G. J., FARWELL, J. R. & FLANNERY, J. T. (1976*a*) Glioblastoma multiforme in children. *J. Neurosurg.*, **44**, 442.

DOHRMANN, G. J., FARWELL, J. R. & FLANNERY, J. T. (1976*b*) Ependymomas and ependymoblastomas in children. *J. Neurosurg.*, **45**, 273.

DOLL, R., PAYNE, P. & WATERHOUSE, J. (1966) *Cancer Incidence in Five Continents. A technical Report.* Berlin: Springer-Verlag.

DOLL, R., MUIR, C. & WATERHOUSE, J. (1970) *Cancer Incidence in Five Continents.* Vol. II. Berlin, Heidelberg, New York: Springer-Verlag.

GEISSINGER, J. D. & BUCY, P. C. (1971) Astrocytomas of the cerebellum in children. Long-term study. *Arch. Neurol.*, **24**, 125.

GJERRIS, F. (1976) Clinical aspects and long-term prognosis of intracranial tumours in infancy and childhood. *Dev. Med. Child. Neurol.*, **18**, 145.

GJERRIS, F., KLEE, J. G. & KLINKEN, L. (1976) Malignancy grade and long-term survival in brain tumours of infancy and childhood. *Acta Neurol. Scand.*, **53**, 61.

GOL, A. (1962) Cerebral astrocytomas in childhood. A clinical study. *J. Neurosurg.*, **19**, 577.

GOL, A. (1963) Cerebellar astrocytomas in children. *Am. J. Dis. Child.*, **106**, 21.

HEISKANEN, O. (1977) Intracranial tumors of children. *Childs Brain*, **3**, 69.

HENDRICK, E. B., HOFFMAN, H. J. & HUMPREYS, R. P. (1975) Treatment of infratentorial gliomas in childhood. In *Gliomas.* Ed. J. Hekmatpanah. Berlin: Springer-Verlag.

HESHMAT, M. Y., KOVI, J., SIMPSON, C., KENNEDY, J. & FAN, K. J. (1976) Neoplasms of the central nervous system. Incidence and population selectivity in the Washington DC, metropolitan area. *Cancer*, **38**, 2135.

KOOS, W. T. & MILLER, M. H. (1971) *Intracranial Tumours of Infants and Children.* Stuttgart: G. Thieme.

KRENKEL, W. (1972) Indikationen un Ergebnisse neurochirurgischer Eingriffe bei Hirntumoren im Kindesalter. *Med. Welt.*, **23**, 589.

LASSITER, K. R. L., ALEXANDER, E., DAVIS, C. H. & KELLY, D. L. (1971) Surgical treatment of brain stem gliomas. *J. Neurosurg.*, **34**, 719.

MARSDEN, H. B. & STEWARD, J. K. (1968) *Tumours in children. Recent Results in Cancer Research.* Vol. 13. Berlin, New York: Springer-Verlag.

MATSON, D. D. (1969) *Neurosurgery of Infancy and Childhood.* 2nd ed. Springfield, Ill.: Charles C. Thomas.

MCFARLAND, D. R., HORWITZ, H., SAENGER, E. L. & BAHR, G. K. (1969) Medulloblastoma—a review of prognosis and survival. *Br. J. Radiol.*, **42**, 198.

ODOM, L. P., DAVIS, C. H. & WOODHALL, B. (1956) Brain tumors in children. *Pediatrics*, **18**, 856.

PERCY, A. K., ELVEBACK, L. R., OKAZAKI, H. & KURLAND, L. T. (1972) Neoplasms of the central nervous system. Epidemiologic considerations. *Neurology (Minneap.)*, **22**, 40.

RUSSELL, D. S., RUBINSTEIN, L. J. (1977) *Pathology of Tumours of the Nervous System.* 4th Ed. London: Edward Arnold.

SCHOENBERG, B. S., SCHOENBERG, D. G., CHRISTINE, B. W. & GOMEZ, M. R. (1976) The epidemiology of primary intracranial neoplasms of childhood. *Mayo Clin. Proc.*, **51**, 51.

SLOOFF, A. C. J. & SLOOFF, J. L. (1975) Supratentorial tumours in children. In *Handbook of Clinical Neurology. Tumours of the Brain and Skull.* Vol. 18. Ed. P. J. Vinken and G. W. Bruyn. Amsterdam: North Holland.

STATISTISK AARBOG (1975) *Statistical Yearbook of Denmark.* Copenhagen: Danmarks Statistik.

STEWART, A. M., LENNOX, E. L. & SANDERS, B. M. (1973) Group characteristics of children with cerebral and spinal cord tumours. *Br. J. Cancer*, **28**, 568.

TEPPO, L., SALONEN, T. & HAKULINEN, T. (1975) Incidence of childhood cancer in Finland. *J. Natl. Cancer Inst.*, **55**, 1065.

TILL, K. (1975) *Paediatric Neurosurgery for Paediatricians and Neurosurgeons.* Oxford: Blackwell.

WEICKMANN, F. (1969) Prognose intrakranieller

Tumoren des Kindesalters. *Zbl. Neurochir.*, **30**, 227.
YATES, A. J. & BECKER, L. E. (1976) A statistical analysis of 704 childhood tumors of the nervous system. *J. Neuropathol. Exp. Neurol.*, **35**, 363.
YOUNG, J. L. & MILLER, R. W. (1975) Incidence of malignant tumors in U.S. children. *J. Pediatr.*, **86**, 254.
ZÜLCH, K. J. (1965) *Brain Tumours, their Biology and their Pathology*. 2nd ed. New York: Springer.
ZÜLCH, K. J. (1971) *Atlas of the Histology of Brain Tumors*. Berlin, New York: Springer-Verlag.